# Enhanced Piezoelectric Properties of Poly(Vinylidenefluoride-Co-Trifluoroethylene)/Carbon-Based Nanomaterial Composite Films for Pressure Sensing Applications

**DOI:** 10.3390/polym12122999

**Published:** 2020-12-16

**Authors:** Jia-Wun Li, Chen-Yang Huang, Kuan-Yu Chen, Jian-Xun Chen, Xiao-Yong Hsu, Yan-Feng Chen, Chung-Feng Jeffrey Kuo, Chih-Chia Cheng, Maw-Cherng Suen, Chih-Wei Chiu

**Affiliations:** 1 Department of Materials Science and Engineering, National Taiwan University of Science and Technology, Taipei 10607, Taiwan; a12352335@yahoo.com.tw (J.-W.L.); D10504015@mail.ntust.edu.tw (C.-Y.H.); M10704205@mail.ntust.edu.tw (K.-Y.C.); ch60210@gmail.com (J.-X.C.); xyhsu.0879@ttri.org.tw (X.-Y.H.); kk0960216886@gmail.com (Y.-F.C.); jeffreykuo@mail.ntust.edu.tw (C.-F.J.K.); 2 Graduate Institute of Applied Science and Technology, National Taiwan University of Science and Technology, Taipei 10607, Taiwan; cccheng@mail.ntust.edu.tw; 3 Department of Fashion Business Administration, Lee-Ming Institute of Technology, New Taipei City 24305, Taiwan; sunmc0414@gmail.com

**Keywords:** piezoelectric, poly(vinylidenefluoride-co-trifluoroethylene, carbon black, carbon nanotubes, graphene oxide

## Abstract

In this study, heat and polarization treatments were applied to poly(vinylidenefluoride-co-trifluoroethylene (PVDF-TrFE) films to improve their crystallinity and piezoelectric effect. Carbon-based nanomaterials (CBNs) of multiple dimensions (i.e., modified zero-dimensional (0D) carbon black (OCB), one-dimensional (1D) modified carbon nanotubes (CNT–COOH) and two-dimensional (2D) graphene oxide (GO)) were added to the copolymer to study the effects of different CBN dimensions on the crystallinity and piezoelectric effect of PVDF-TrFE films. Additionally, amphiphilic polymeric dispersants were added to improve the dispersibility of CBNs; the dispersant was synthesized by the amidation, and imidization reactions of styrene-maleic anhydride copolymer (SMAz) and polyoxyalkylene amine (M1000). Polymer solutions with different ratios of CBN to dispersant (z = 10:1, 5:1, 1:1, 1:5, 1:10) were prepared. The enhanced dispersibility enabled the fluorine atoms in the PVDF-TrFE molecular chain to more efficiently form hydrogen bonds with the –COOH group in the CBN, thereby increasing the content of the β crystal phase (the origin of the piezoelectric effect) of the film. Therefore, the resulting film exhibited a higher output voltage on the application side and better sensitivity on the sensing element. The addition of CNT–COOH and polymeric dispersants increased the β-phase content in PVDF-TrFE from 73.6% to 86.4%, which in turn raised the piezoelectric coefficient from 19.8 ± 1.0 to 26.4 ± 1.3 pC/N. The composite film-based pressure sensor also exhibited a high degree of sensitivity, which is expected to have commercial potential in the future.

## 1. Introduction

Recent years have witnessed a consistent rise in public demand for electronic products [1], among which wearable devices have gained attention for their comfort, flexibility, and durability [2,3]. Meanwhile, in order to reduce global energy consumption, the development of alternative energy continues to gain traction [4]. The renewable energies currently under development include solar, thermal, wind, and kinetic energies [5,6]. Three methods are available for harvesting vibration energy, namely, electrostatic, electromagnetic, and piezoelectric methods [7]. The ceramic piezoelectric materials are generally characterized by high-voltage and high-dielectric properties. However, their applications are still limited because of various deficiencies. For example, they are too hard and brittle, difficult to process, and bio-incompatible [8]. Piezoelectric polymers, with advantages such as simple processing routes, light, and thin structures, durability, high chemical resistance, low environmental sensitivity, and biocompatibility, boast considerable potential in wearable devices [9]. Polyvinylidene (PVDF) [10] and poly(vinylidenefluoride-co-trifluoroethylene (PVDF-TrFE) [11] are among the materials that have attracted significant attention from the academic world in recent years because they are scratch-resistant, easy to process, and structurally light and thin [12]. The crystalline forms of both PVDF and PVDF-TrFE are composed of a combination of α- and β-phases; the α-phase is most commonly seen in PVDF or its copolymers, but the β-phase is more important to the piezoelectric effect because the β-phase has both piezoelectric and electrothermal properties [13]. Additionally, the mechanism underpinning the induced α to β-phase transformation in PVDF and its copolymers has been widely studied [14,15,16]. Specific intermolecular forces (van der Waals forces, ion coupling, and hydrogen bonding) can be used to induce the α- to β-phase transformation in PVDF and its copolymer [17,18,19].

For example, Shuai [20] and Bhavanasi et al. [21] respectively used oxygen-containing groups on the surface of graphene oxide and hydrogen bonding between PVDF-TrFE to induce the crystallization of the ferroelectric β-phase, which exhibited good performance in electrical output and energy harvesting devices. In terms of piezoelectric efficiency, although PVDF has a higher piezoelectric coefficient (d_33_, 20–28 pC/N) than PVDF-TrFE. Levi et al. [22] nevertheless showed that, compared with PVDF, the use of the PVDF-TrFE copolymer could more effectively facilitate the dispersion of nanocarbon materials in solution. Meanwhile, the interaction between the functional groups on the surface of the nanomaterial and PVDF (or its copolymers) can be harnessed to promote the β-phase [23], thereby improving the crystallinity and piezoelectric properties of PVDF (or its copolymers). In our laboratory, we have comprehensively studied the dispersion and stabilization of inorganic nanomaterials by polymeric dispersants [24,25,26,27]; the molecular structure of the polymeric dispersant has been used to interact with the inorganic nanomaterial via π–π stacking and lone pair interactions, thereby enhancing the dispersion and stability of the inorganic nanoparticle in solution. To our knowledge, no research has discussed the seismocardiography influence of carbon nanomaterials of different dimensions on piezoelectric polymers. Previously reported [28] using nonlocal continuum methods to evaluate the structural behavior of nano-sensors and nano-actuators on nano-beams such as strain gradient method and stress driven theory. The models provide a stiffening structural behavior based on experimental evidence [29].

In this study, oxidized carbon black (OCB) with carboxylic acid or hydroxyl groups on the surface, modified carbon nanotubes (CNT–COOH), and graphene oxide (GO) were added to the PVDF-TrFE solution in different weight ratios, after which a composite piezoelectric film of carbon nanomaterial/PVDF-TrFE (nanocarbon/PVDF-T) was prepared. The addition of carbon nanomaterials can induce a higher proportion of the β-phase structure and improve the overall piezoelectricity and mechanical strength of the film. As the high surface energy of the nanoparticles may cause agglomeration and therefore worsen the resultant properties of the material, styrene maleic acid-amide (SMA-amide) was added as a dispersant to improve the dispersion of the modified carbon nanotubes in the PVDF-TrFE solution; better piezoelectric effects were generated by improving the dispersion of the carbon materials in the film. Finally, the composite film was applied in seismocardiography (SCG) physiological signal sensors, pressure sensors, and energy harvesting devices.

## 2. Experimental

### 2.1. Materials

Poly(vinylidenefluoride-co-trifluoroethylene (PVDF-TrFE, Tc = 127.4 °C, Tm = 148.8 °C) was procured from Piezotech (Pierre-Benite, France). Carbon black (CB, particle size 40 nm) and oxidized carbon black (OCB, oxygen content ~20%, particle size 40 nm) were purchased from MTI Japan (Yokohama). Multi-walled carbon nanotubes (CNT, diameter 10–20 nm), carboxyl group enriched multi-walled carbon nanotubes (CNT–COOH, diameter 10–20 nm, oxygen content ~25%), reduced graphene oxide (rGO, diameter 550 nm, oxygen content 4~7%), and graphene oxide powder (GO, film diameter 550 nm, oxygen content 50%) were purchased from Conjutek Ltd (Taipei, Taiwan). Styrene maleic anhydride (SMA 1000, SMA 2000, SMAEF 40, SMAEF 80) was purchased from Yuang Hong Corp. (Taipei, Taiwan) and Jeffamine M1000 (M1000) was procured from Huntsman Corporation (Salt Lake City, UT, USA).

### 2.2. Preparation of Carbon Nanomaterial/Poly(Vinylidenefluoride-Co-Trifluoroethylene) Composite Piezoelectric Film

Methyl ethyl ketone (MEK) was added to reduce the boiling point of the solvent; thus, the solvent could be completely volatilized below the melting point of PVDF-TrFE. PVDF-TrFE with a weight concentration of 20 wt% was then added to the solvent, and the mixture was stirred with a heated stirrer at 50 °C for 2 h to ensure dissolution; thus a PVDF-TrFE solution was obtained. Then, the carbon material was added in different concentrations with respect to the solvent (0.1 wt%, 0.5 wt%, 1 wt%, 3 wt%, or 5 wt%). The mixture was ultrasonicated for 20 min to physically disperse the carbon nanomaterials. The dispersed material was then added to a pre-configured PVDF-TrFE solution to prepare a carbon nanomaterial/PVDF-TrFE (nanocarbon/PVDF-T) solution. Next, the nanocarbon/PVDF-T solution was wet coated onto a high-temperature resistant glass, placed in a vacuum oven for defoaming, and then dried at 80 °C for 2 h to completely evaporate the solvent, producing a nanocarbon/PVDF-T composite film of approximately 80 µm in thickness. At this stage, the film mostly contained the α-phase, which does not have piezoelectric properties (i.e., an absence of the piezoelectric effect). Subsequent annealing and polarization treatments were needed to transform the α-phase into the β-phase, which has piezoelectric characteristics. Therefore, the nanocarbon/PVDF-T composite film was placed in a vacuum oven at various temperatures of an annealing process for 4 h. Next, the annealed film was coated with silver glue on both sides to add the conductive layers. The film was then placed in a vacuum oven for 1 h until the solvent in the silver glue completely evaporated. The film was then taken out and polarized at a temperature of 50 °C under different high-voltage electric fields for 2 h to finalize the preparation for the nanocarbon/PVDF-T composite piezoelectric film.

### 2.3. Synthesis of Styrene Maleic Anhydride (SMA)-Amide

SMA-amide was synthesized based on the practice previously adopted in the laboratory [30]. Four types of styrene maleic anhydride copolymers (SMAz; z = 1000, 2000, EF40, EF80) with different benzene ring numbers were used in an amination grafting reaction with polyether monoamine (M1000, polyetheramine) in a molar ratio of 1:1 at 25 °C with tetrahydrofuran (THF) as the organic solvent, from which the star-shaped oil-soluble dispersant styrene maleic acid-amide (SMAz-M) was generated. First, a 50 mL three-necked round-bottom flask was fitted with a magnetic stirrer, nitrogen inlet/outlet tube, and a thermometer as the experimental instruments; 0.005 mol of SMAz (z = 1000, 2000, EF40, EF80) was added to 5 mL of THF. Then, 5 mL of THF containing 0.005 mol of M1000 was prepared, and the solution was titrated into the round bottom flask through a funnel. The mixture was vigorously stirred and kept at 25 °C for 4 h and then heated to 80 °C for 4 h for the reaction. Finally, the THF solvent was volatilized to obtain SMAz-M (z = 1000, 2000, EF40, EF80).

### 2.4. Preparation of Composite Piezoelectric Film with Modified Carbon Nanotube Dispersion

The four dispersants, SMAz-M (z = 1000, 2000, EF40, EF80), were added to disperse the modified carbon nanotubes (CNT–COOH) and to achieve improved stability in solution. The carbon nanomaterial (0.005 g) was added to the 1:1 DMF:MEK co-solvent (5 g), and the mixture was ultrasonicated for 20 min. The nanocarbon material was completely dispersed in the solution after the mixture was stirred for 10 min in an ultrasonic cell crusher. Then, SMAz-M was added in the weight ratio (nanocarbon material:SMAz-M) of 10:1, 5:1, 1:1, 1:5, and 1:10, and a heated stirrer was used to stir the mixture at 50 °C for 2 h to evenly mix the modified carbon nanotube dispersion with the PVDF-TrFE solution to achieve good dispersibility. After stirring, the modified carbon nanotube/PVDF-T solution will generate tiny bubbles in the solution. To avoid the generation of pores in the subsequent production of the film, which will affect the crystallinity and piezoelectric effect, the solution was ultrasonically shaken and then placed in a vacuum oven for defoaming so that the pores were inhibited during the film formation process.

### 2.5. Characterization and Instruments

X-ray diffraction (XRD; D2 Phaser, Bruker, Karlsruhe, Germany) was used to characterize PVDF-TrFE (after the annealing treatment at different temperatures, a polarization treatment at different voltage) and to analyze the crystallinity of the (OCB, CNT–COOH, GO)/PVDF-T composite piezoelectric film. The sample was cut to a size of 3 cm × 3 cm and placed in a vacuum oven. When it was completely dried, testing was then conducted (θ range: 10–50°, 0.5 s for each point). Fourier transform infrared spectroscopy (FTIR; FTS-1000, Digilab, Hopkinton, MA, USA) was used to identify the functional groups of the carbon material before and after modification and to calculate the percentage of the β-phase of the composite piezoelectric film. The nanocarbon powder and piezoelectric composite material were placed on a contact platform and scanned within the range 400–4000 cm^−1^ at a resolution of 2 cm^−1^. Differential scanning calorimetry (DSC; DSC 6000, Perkin Elmer, Waltham, MA, USA) was used to characterize the thermal properties of the materials. The sample was packaged in an aluminum tray with a lid, heated to 180 °C to eliminate the thermal history, then cooled to 60 °C at a rate of −10 °C/min, and finally heated to 180 °C at a rate of 10 °C/min where the second heating thermogram was recorded. The cross-section of the piezoelectric composite material was frozen in liquid nitrogen to fix its structure. Then, the sample was cut in half and plated with platinum before it was placed under a high-resolution field-emission scanning electron microscope (FESEM; JSM-6500F, JEOL, Tokyo, Japan). A UV–Visible spectrophotometer (UV–Vis; V-630, Jasco Corp., Kyoto, Japan)) was used to measure the dispersibility of the modified carbon nanotubes (CNT–COOH) in SMAz-M (z = 1000, 2000, EF40, EF80) at 550 nm. A transmission electron microscope (TEM; EM 902A, Zeiss, Oberkochen, Germany) was used to identify the dispersion at different ratios (10:1, 5:1, 1:1, 1:5, 1:10) of the modified carbon nanotubes (CNT–COOH) to SMAz-M (z = 1000, 2000, EF40, EF 80). The sample solution was dripped onto the carbon-plated copper mesh at a weight concentration of 0.01 wt% before it was placed in an oven to completely evaporate the solvent, and the resulting sample was examined for the dispersion of the nanocarbon material. For the voltage test, a universal tensile machine (MTS-370, MTS Systems Corp., Eden Prairie, MN, USA) equipped with a compression clamp was employed. The sample was cut into a voltage test pressure sheet with a diameter of 30 mm and then placed between the upper and lower clamps with a configuration of 200 N, 300 N, and 400 N with the frequency set to one per second; when the sample was pressed, an oscilloscope (PicoScope 4224, Pico Technology Ltd., Cambridgeshire, UK) was used to receive the voltage value generated by the composite piezoelectric film. Contact angle measurements, performed using deionized water on samples, were recorded using a Sindatek instrument (model 100SB, Sindatek Instruments Co. Ltd., New Taipei, Taiwan) at room temperature. Raman spectra were recorded and integrated with a HORIBA iHR550 Raman microscope system (Protrustech Corp. Ltd., Tainan, Taiwan), and the light detection used a silicon CCD camera. It was focused via a 50× objective lens (Olympus BX-41) onto the sample with a laser operating at λ = 532 nm.

## 3. Results and Discussion

### 3.1. Poly(vinylidenefluoride-co-trifluoroethylene (PVDF-TrFE) Annealing Process and Polarization Treatment

**Figure 1:**
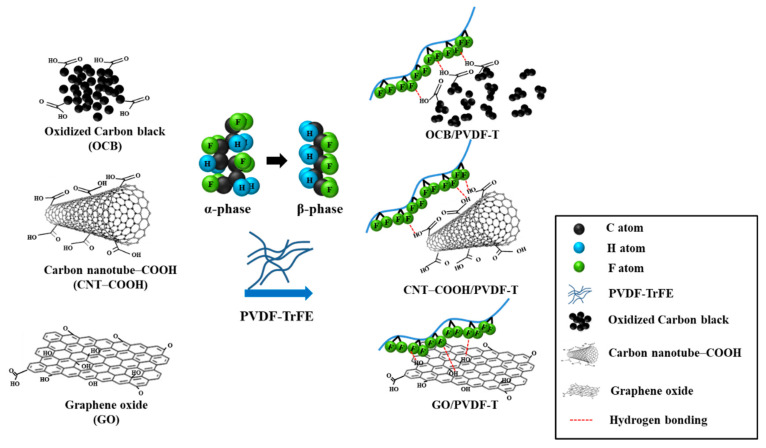
The schematic of carbon nanomaterials inducing the transformation of the α-phase to β-phase of PVDF-TrEE.

The main aim of this study was to use the carboxylic acid or hydroxyl groups on the surface of OCB, CNT–COOH, and GO to form hydrogen bonds with the fluorine element in the PVDF-TrFE film to induce the formation of the β-phase structure, as depicted in Figure 1. As a result of this process, the PVDF-TrFE piezoelectric films containing the carbon nanomaterials were superior to the original film in terms of piezoelectric properties. Therefore, it was necessary to identify the best processing conditions for the PVDF-TrFE piezoelectric film.

**Figure 2:**
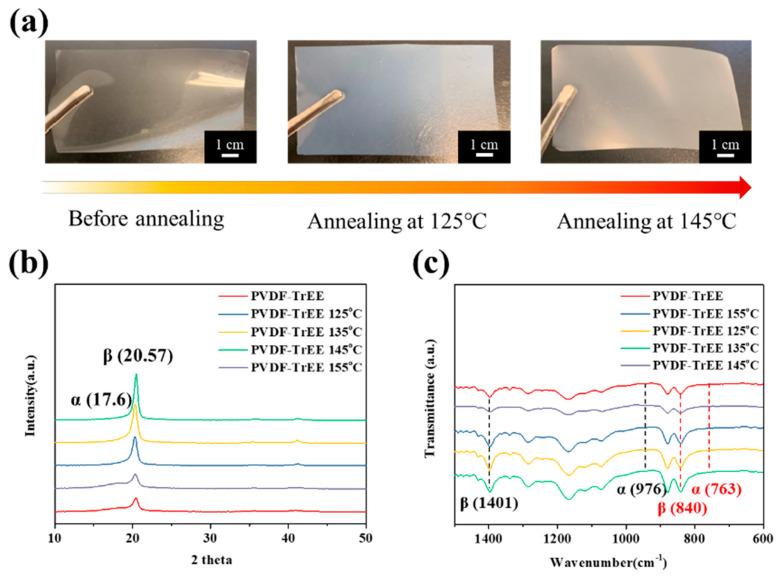
(**a**) Appearance, (**b**) XRD patterns, and (**c**) FTIR curves of PVDF-TrEE after different annealing temperatures.

First, the original PVDF-TrFE film was annealed. As shown in Figure 2a, after the PVDF-TrFE film was annealed at 125 °C and 145 °C, the exterior of the film gradually changed from a transparent film to an opaque film. This is because the PVDF-TrFE molecular chain structure was transformed from the α-phase to the β-phase. Then, XRD and FTIR were used to calculate the changes in the crystallinity of PVDF-TrFE annealed at different temperatures, as shown in Figure 2b,c. From the XRD patterns, it can be seen that when the annealing temperature reached 145 °C, the crystallinity of the PVDF-TrFE film gradually increased, and when the temperature increased to 155 °C, the crystallinity of the film significantly decreased. This is because the temperature of the annealing process usually needs to be above the recrystallization temperature (Tc) and below the melting temperature (Tm) of the film, and the annealing temperature exceeded the melting point of PVDF-TrFE (Tm = 155 °C), which significantly reduced the crystallinity.

The β/β_0_ and crystallinity of PVDF-TrFE after annealing at different temperatures was then calculated, as shown in Table 1. Compared to the original state, the crystallinity of untreated PVDF-TrFE increased from 56.8% to 71.9% after annealing at 145 °C, and the area of the β-phase peak at 2θ = 20.57° increased by 131.7%; importantly, the α-phase peak at 2θ = 17.6° disappeared after annealing. The FTIR spectra showed that the characteristic peaks of the α-phase occurred at the wavelengths of 763 cm^−^^1^ and 976 cm^−^^1^, and the characteristic peaks of the β-phase occurred at 840 cm^−^^1^ and 1401 cm^−^^1^. The crystallinity of the β-phase (F_(β)_) can be calculated by using the Beer–Lambert Law as expressed in Equation (1):

(1) $$F_{\beta }=\frac{A_{\beta }}{(\frac{K_{\beta }}{K_{\alpha }}{)A}_{\alpha }{+A}_{\beta }}=\frac{A_{\beta }}{1.3A_{\alpha }{+A}_{\beta }}$$

where K_α_ and K_β_ are the molar area coefficients of the PVDF-TrFE film, which are 6.1 × 10^4^ cm^2^ mol^−^^1^ and 7.7 × 10^4^ cm^2^ mol^−^^1^, respectively; A_α_ is the area of the characteristic peak of the α-phase at 763 cm^−^^1^; and A_β_ is the area of the characteristic peak of the β-phase at 840 cm^−^^1^. The calculation results are summarized in Table 1, which shows that annealing at 145 °C increased the crystallinity of the β-phase from 62.1% to 69.5%, while the crystallinity of the β-phase decreased to 55.6% when the melting point of PVDF-TrFE was exceeded, which is consistent with the XRD analysis results. Similarly, the decrease in the transparency of the film as the annealing temperature approached 145 °C (Figure 2a) can be attributed to the gradual rise in the crystallinity of the film. Overall, the results revealed that annealing at 145 °C produced the greatest increase in the crystallinity of the film. Subsequently, the PVDF-TrFE film was polarized by the hot electrode method to increase the crystallinity. A positive voltage was applied to one side of the film, which was then placed on a conductive platform connected to a negative voltage, and high voltages of different values were applied to the film at 50 °C. High voltages of 1000, 2000, 3000, 4000, and 5000 V were applied at 50 °C to observe the changes in the crystallinity of the PVDF-TrFE film, as shown in Figure S1a. The crystallization calculations are summarized in Table 1; the results revealed that with increasing voltage, the β-phase crystallinity percentage of the PVDF-TrFE film gradually increased, but the β-phase crystallinity of the film at 5000 V and 4000 V did not increase significantly. A further examination of the hysteresis curve of the PVDF-TrFE film after annealing at 145 °C (as shown in Figure S1b) shows that the curve converged at approximately 50 V/µm. Given that the film thickness of the PVDF-TrFE film was 80 µm, calculations show that the PVDF-TrFE film only needed approximately 4000 V (50 V/µm) to achieve polarization of the dipole moments. Therefore, to avoid the electrical breakdown caused by excessive voltage, which could lead to defects on the surface of the material and thus affect the piezoelectric effect of the film, a relatively low voltage (4000 V) was selected for subsequent polarization experiments. Finally, the piezoelectric coefficient (d_33_) in the z-axis direction was recorded for the untreated PVDF-TrFE film, the PVDF-TrFE film annealed at 145 °C, and the PVDF-TrFE film annealed at 145 °C and polarized at 4000 V; these results are summarized in Table 1. The best reference data for the piezoelectric effect of most piezoelectric materials were d_33_, which refers to the Coulombs of current generated per Newton force. After the film was annealed and polarized, the application of 1 N force along the z-axis should generate a current of 19.8 ± 1.0 °C. The results showed that the annealing treatment did not significantly improve the piezoelectric coefficient; however, the piezoelectric coefficient of the film improved significantly after the polarization treatment. This is because the dipole moments of the film were non-directionally arranged before polarization and canceled each other, whereas after the polarization treatment, the dipole moments were arranged in a consistent direction; therefore, voltage was generated when the film was subjected to an external force.

### 3.2. Carbon Nanomaterials/PVDF-TrFE Piezoelectric Composite Film

**Figure 3:**
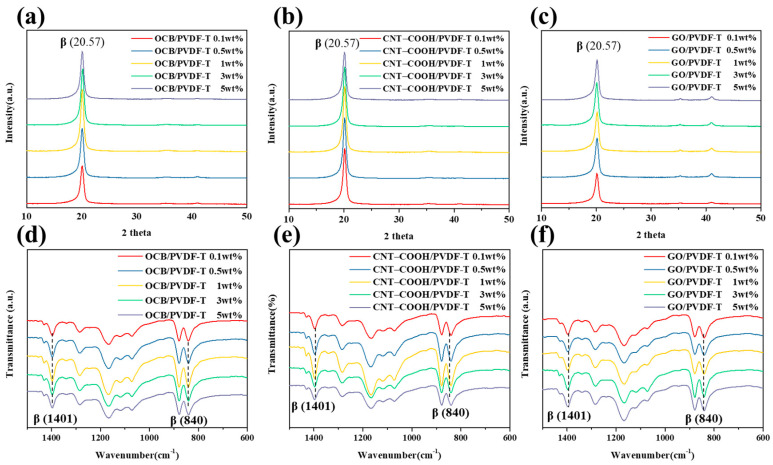
(**a**–**c**) XRD and (**d**–**f**) FTIR curves of PVDF-TrEE with different ratios of OCB, CNT–COOH, and GO.

To facilitate the α- to β-phase transformation of PVDF-TrFE, inorganic substances containing functional groups were incorporated into the polymer matrix. In this study, the hydrogen bond between the fluorine atoms of PVDF-TrFE and the hydrogen atoms of the carboxylic acid or hydroxyl groups on the surface of the inorganic substance were leveraged to induce PVDF-TrFE to form more β-phases. As shown in Figure S2, when the inorganic carbon nanomaterials were oxidized, their –OH and –COOH functional groups increased significantly, thereby increasing the probability of β-phase formation. On the other hand, to avoid significant changes in the annealing temperature after the inorganic substances were added to PVDF-TrFE, Figure S3 shows the DSC graphs of PVDF-TrFE mixed with different oxidized nanocarbon materials. As shown in Figure S3, when PVDF-TrFE was mixed with oxidized carbon nanomaterials of different dimensions, the crystallization temperature remained at approximately 127 °C, and the melting point was maintained within the range of 149–152 °C; therefore, the annealing temperature was maintained below 145 °C, at which the PVDF-T composite film was annealed. Subsequently, the results indicated that the melting enthalpy (ΔHc) of PVDF-TrFE, OCB/PVDF-T 5 wt%, CNT–COOH/PVDF-T 5 wt%, and GO/PVDF-T 5 wt% were 21.6, 22.5, 34.5, and 25.6 J/g after calculating the melting enthalpy, indicating that the crystallinity of PVDF-TrFE can be effectively improved when carbon nanomaterials are added to PVDF-TrFE. Figure 3a–c shows the XRD patterns of the nanocarbon/PVDF-T composite films with different dimensions and different addition amounts of the carbon nanomaterial, and the calculations of crystallinity are shown in Table 2. The results demonstrate that with 1 wt% OCB, the film crystallinity reached 83.1%, and with 3 wt% OCT, the crystallinity decreased; in the CNT–COOH system, with 1 wt% addition, the film crystallinity reached 83.4%, and with 3 wt% addition, the crystallinity decreased; in the GO system, with 3 wt% addition, the degree of film crystallinity reached 76.6%, and with 5 wt% addition, the crystallinity decreased. This may be because an excessive concentration of the carbon material deteriorates the polymer matrix or decreases the dispersibility of the nanomaterial. In addition, the optimal addition amount of OCB and CNT–COOH was found to be 1 wt%, while that of GO was 3 wt%. This difference in the optimal amount may arise from the difference in the oxygen content of the carbon nanomaterials. For OCB and CNT, the oxygen content of –COOH was respectively 20% and 25%, while the oxygen content of GO was as high as 50%. Therefore, when GO was mixed into PVDF-TrFE, the greater number of –OH and –COOH functional groups on the surface of GO produced more hydrogen bonds with fluorine atoms, resulting in a good dispersion effect at higher concentrations. Figure 3d–f shows the FTIR spectra of the nanocarbon/PVDF-T composite films of different dimensions and different additions of the carbon nanomaterial. The characteristic peaks of the α- and β-phases were observed in all the spectra.

**Figure 4:**
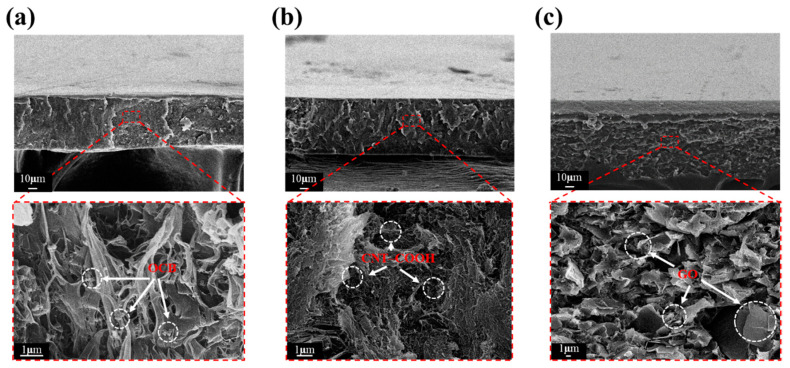
Scanning electron microscopy (SEM) cross-sectional images of the piezoelectric composite films: (**a**) OCB/PVDF-T 1 wt%, (**b**) CNT–COOH/PVDF-T 1 wt%, and (**c**) GO/PVDF-T 3 wt%.

Despite the continuous efforts invested in seeking optimal parameters, as shown in Figure 4, it is nevertheless difficult to avoid the agglomeration of trace inorganic substances without the addition of any dispersant because of the intrinsic properties of inorganic substances. This kind of micro-agglomeration slightly affects the piezoelectric properties of the piezoelectric film. Therefore, this study adopted the previous practice of the laboratory to synthesize SMA-M dispersants and added them to the 1 wt% CNT–COOH/PVDF-T solution, in the hope that the piezoelectric performance can be further improved.

The crystallinity of the β-phase was then calculated according to the Beer–Lambert Law, and the results are shown in Table 2**.** The optimal addition amounts of the three nanocarbon materials were 1 wt% for OCB, 1 wt% for CNT–COOH, and 3 wt% for GO, and the β-phase crystallinity for these additions were 80.9%, 82.8%, and 77.3%, respectively. After the optimal addition amounts for each carbon nanomaterial were identified, the piezoelectric coefficients (d_33_) in the z-axis direction were measured for the three different nanocarbon/PVDF-T composite films, and the results are summarized in Table 2**.** The d_33_ of OCB/PVDF-T 1 wt%, CNT–COOH/PVDF-T 1 wt%, and GO/PVDF-T 3 wt% were 23.2 ± 1.4, 24.3 ± 1.3, and 20.6 ± 1, respectively. The overall results (XRD, FTIR, and piezoelectric coefficients) showed that CNT–COOH/PVDF-T at 1 wt% exhibited the greatest crystallinity and piezoelectric effect. This may be attributed to the similarity in the one-dimensional structure of carbon nanotubes and the molecular chain of the β-phase of PVDF-TrFE, and as a result of this similarity, it was easier for hydrogen bonds to form between the fluorine atoms in the PVDF-TrFE molecular chain and the hydrogen atoms on the surface of the carbon nanotubes, thereby promoting the α- to β-phase transformation. 

### 3.3. Dispersion Stability of CNT–COOH Using Polymeric Dispersant

**Figure 5:**
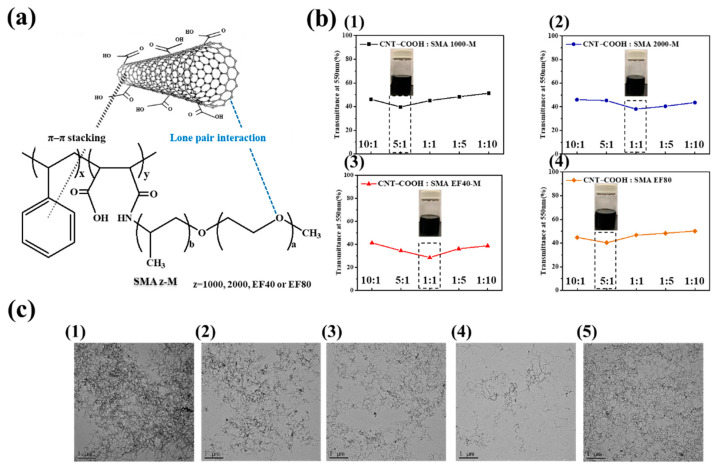
(**a**) Schematic of the dispersion mechanism of CNT–COOH by polymeric dispersant. (**b**) Transmittance of CNT–COOH with different polymeric dispersants in different proportions at a wavelength of 550 nm. (**c**) TEM images of (**1**) no polymeric dispersant added, (**2**) CNT–COOH:SMA1000-M 5:1, (**3**) CNT–COOH:SMA2000-M 1:1, (**4**) CNT–COOH:SMAEF40-M 1:1, (**5**) CNT–COOH:SMAEF80-M 5:1.

The polymeric dispersant was made by combining four different styrene maleic anhydride copolymers (SMA 2000, SMA 1000, SMA EF40, and SMA EF80) with polyether monoamine (Jeffamine M1000) in different ratios at 25 °C to prepare styrene maleic acid-amide (SMAz-M), a dendritic lipophilic polymeric dispersant; the differences in the number of benzene rings were harnessed to examine the dispersion of CNT–COOH in the solvent with different dispersants. The chemical reaction of the polymeric dispersants is shown in Figure S4, and the reaction process was monitored using FTIR, as shown in Figure S5. The FTIR bands at 3093–2848 cm^−1^ and 1495–1450 cm^−1^ are assigned to =C–H and C=C stretching of aromatic benzene ring, respectively. The anhydride (C=O) stretching vibration peaks of SMA EF40 occur at 1855 cm^−1^ and 1780 cm^−1^ [31]. After the amination reaction, the anhydride stretching vibration peak disappeared in the FTIR spectrum of SMAEF40-M, while the classical amide absorption peaks (–CONH) appeared at 1652 cm^−1^ and 1550 cm^−1^. In addition, at a frequency of 3726–3130 cm^−1^, a broad peak at the –OH stretching vibration peak of carboxylic acid appears, indicating that SMA EF40 and Jeffamine M1000 completed the amide reaction, and a similar change in FTIR was consistent with previous reports [30]. Figure 5a shows the mechanism by which the polymeric dispersant improves the dispersion and stability of modified carbon nanotubes (CNT–COOH). Both the lipophilic benzene ring of the dispersant and the aromatics on the modified carbon nanotubes engage in π–π stacking. Additionally, lone pair interactions exist between the CNT–COOH and the dispersant. These intermolecular interactions serve to increase the spacing between the carbon nanotubes, thereby restraining their agglomeration. Furthermore, the good solubility of the organic dispersants in solvents can be harnessed to enhance the dispersion and stability of carbon nanomaterials in solvents [24]. CNT–COOH was dispersed in a solvent mixed with a polymeric dispersant; the mixed solutions contained different weight ratios (10:1, 5:1, 1:1, 1:5, and 1:10) of CNT–COOH to polymeric dispersant. A transmittance analysis of the UV–Vis spectrum revealed that the absorption peak of CNT–COOH occurred at a wavelength of 550 nm; therefore, the wavelength of 550 nm was used in the UV–Vis spectrum to analyze the transmittance of the CNT–COOH dispersion. The lower the transmittance, the more difficult it is for light to penetrate the sample solution; thus, the dispersion effect is improved when the transmittance is low. According to the results shown in Figure 5b, the best CNT–COOH:SMAz-M ratios for z = SMA2000-M, SMA1000-M, SMAEF40-M, and SMAEF80-M were 5:1, 1:1, 1:1, and 5:1, respectively. Figure 5c presents the TEM images of CNT–COOH with no dispersants and with the four dispersants in the best ratio, confirming that the carbon nanotubes without added polymeric dispersants were prone to serious agglomeration and that agglomeration was reduced when a polymeric dispersant was added. This is because the addition of dispersant increased the spacing between the carbon nanotubes, thereby stabilizing the carbon nanotube dispersion. The SMAEF40-M carbon nanotube solution exhibited the best dispersion effect; in the TEM images, the carbon nanotubes were linear with almost no agglomeration and the number of dark black spots was low, indicating that carbon tube clustering was restrained. Additionally, the transmittance analysis and TEM images indicated that the dispersion effect of SMAEF40-M was greatest when the CNT–COOH to SMAEF40-M ratio was 1:1.

### 3.4. CNT–COOH:SMAz-M/PVDF-T Piezoelectric Composite Film

**Figure 6:**
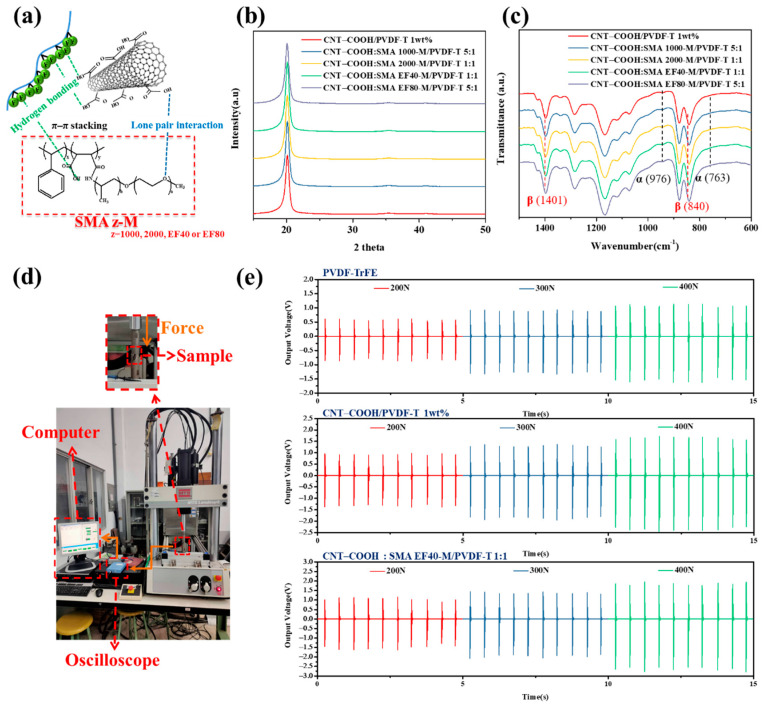
(**a**) Schematic of the polymeric dispersant interacting with CNT–COOH and PVDF-TrFE. (**b**) XRD and (**c**) FTIR curves of CNT–COOH:SMAzM/PVDF-T with different polymeric dispersants at the optimal ratio. (**d**) Photograph of continuous output voltage test setup and (**e**) the continuous output voltage curve of PVDF-TrFE, 1 wt% CNT–COOH/PVDF-T, and 1:1 CNT–COOH:SMA EF40-M/PVDF-T under different pressures.

Figure 6a shows a schematic diagram of the intermolecular forces acting on the polymeric dispersant in CNT–COOH/PVDF-T. In addition to π–π stacking between the polymeric dispersant and the modified carbon nanotubes (CNT–COOH) and the interaction of lone pairs of electrons, the fluorine atoms in PVDF-TrFE could simultaneously form separate hydrogen bonds with the polymeric dispersant and CNT–COOH. Thus, via these intermolecular forces, the polymeric dispersant disperses and stabilizes CNT–COOH and also concurrently induces the formation of the β-phase in PVDF-TrFE. Therefore, the four different polymeric dispersants were added to 1 wt% CNT–COOH/PVDF-T at the optimal ratio, and the resulting crystallinity was measured. Figure 6b,c respectively show the XRD patterns and FTIR spectra of CNT–COOH:SMAz-M/PVDF-T at the optimal ratio; the calculated crystallinity and β-phase percentage are summarized in. The addition of the polymeric dispersant had no obvious effect on the crystallinity of the CNT–COOH/PVDF-T film; the crystallinity was approximately 83.5% with the dispersants and 83.4% without the dispersant. In terms of the β-phase content, the highest percentage (86.4%) was observed for 1:1 CNT–COOH:SMA40-M/PVDF-T, which increased from 82.8% for CNT–COOH/PVDF-T, confirming that the dispersibility of the CNT–COOH/PVDF-T composite piezoelectric film is correlated with the peak value of the β-phase of the film. If the dispersion improves, the OH groups in CNT–COOH can more effectively form hydrogen bonds with the fluorine atoms in the PVDF-TrFE molecular chain. Therefore, the original α-phase molecular chain will transform more effectively into the β-phase to produce a piezoelectric effect. The piezoelectric coefficient (d_33_) of CNT–COOH:SMAEF40-M/PVDF-T 1:1 was determined and compared in Table 2, and the results showed that d_33_ increased from 24.3 ± 1.3 pC/N to 26.4 ± 1.3 pC/N with the addition of the polymeric dispersant, indicating that the polymeric dispersant effectively improved the piezoelectric property of the CNT–COOH/PVDF-T composite piezoelectric film. Subsequently, the water contact angle, Raman spectroscopy, and continuous output voltages of the CNT–COOH:SMA EF40-M/PVDF-T composite piezoelectric film with the highest β-phase crystallinity, CNT–COOH/PVDF-T, and PVDF-TrFE were compared under the same annealing temperature and polarization conditions. Figure S6 displays the water contact angle of the various piezoelectric films. The results revealed the water contact angles of PVDF-TrFE, CNT–COOH/PVDF-T 1 wt%, and CNT–COOH:SMA EF40-M/PVDF-T 1:1 were 80.9, 100.9, and 107.2°. The results indicated that the contact angle to water has a significant upward trend when CNT–COOH was added to PVDF-TrFE because the carbon nanomaterials are distributed on the surface of PVDF-TrFE when PVDF-TrFE was added with CNT–COOH, thereby increasing the surface roughness, which has been reported in previous studies [32,33]. Subsequently, it can be found that the water contact angle increased from 100.9 to 107.2° by adding SMA EF40-M, which may be because SMA EF40-M can improve CNT–COOH dispersal in PVDF-TrFE. Therefore, CNT–COOH was more uniformly dispersed on the surface of PVDF-TrFE and promoted an increase in the contact angle to water. To confirm the effect of SMA-EF40 on CNT–COOH in PVDF-TrFE film, Figure S7 displays the Raman spectroscopy of CNT–COOH/PVDF-T 1 wt% and CNT–COOH:SMA EF40-M/PVDF-T 1:1. The results revealed that the D Band red shifted from 1346.7 to 1346.2 and the G Band red shifted from 1590.0 to 1586.2 when SMA EF40-M was added to CNT–COOH/PVDF-T 1 wt%, There have been reports that have indicated that a red shift phenomenon occurs in the Raman spectrum when CNTs adsorb specific molecules [34], therefore, this phenomenon confirms that a certain interaction was found between SMA EF40-M and CNT–COOH and further promotes the uniform dispersion of CNT–COOH in the PVDF-TrFE film when SMA EF40-M is added to CNT–COOH/PVDF-T 1 wt%. The continuous output voltage under pressure was measured using the compression mold of a universal stretching machine, and the spectrum generated by the computer and the oscilloscope is shown in Figure 6d. Different compression strengths were applied and the continuous voltage outputs were recorded; the results are shown in Figure 6e and the data sorted in Table 3**.** The results showed that pure PVDF-TrFE without additives exhibited poor piezoelectric effect; when the pressure was 200 N, the average voltage of CNT–COOH:SMA EF40-M/PVDF-T was 1.36 V, and the maximum voltage was 1.57 V. When the applied pressure was 400 N, the average voltage reached 2.36 V and the maximum voltage was 2.72 V, representing an increase of 9% compared with the average voltage of 2.16 V and the maximum voltage of 2.50 V of the CNT–COOH/PVDF-T film without a dispersant. Therefore, by improving the dispersibility of the CNT–COOH solution, the enhanced intermolecular interactions between the –COOH groups on the surface of CNT–COOH and the fluorine atoms of PVDF-TrFE promoted a higher proportion of the PVDF-TrFE molecular chain to convert from the α-phase to the β-phase, thereby increasing the output voltage of the film.

### 3.5. Application of the CNT–COOH:SMAEF40-M/PVDF-T Piezoelectric Composite Film

**Figure 7:**
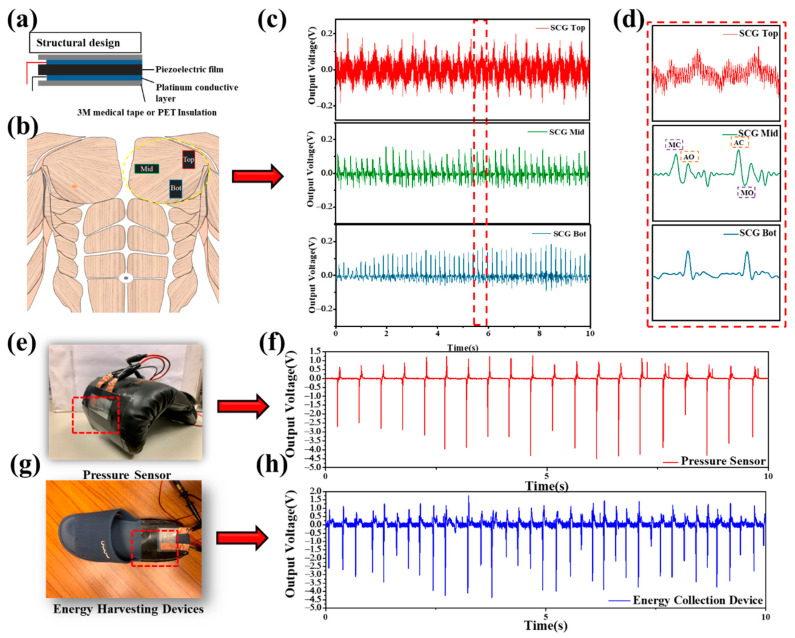
(**a**) Schematic of the CNT–COOH:SMA EF40-M/PVDF-T packaged sensing element, (**b**) schematic of the seismocardiography measurement positions, (**c**) measurement seismocardiography curves at different positions, and (**d**) partial enlarged view of the seismocardiography measurement at different positions. (**e**) Schematic of the pressure sensor and (**f**) measurement curves of the pressure sensor. (**g**) Schematic of the energy collector and (**h**) measurement curves of the energy collector.

According to the results of the above experiments, the CNT–COOH:SMA EF40-M/PVDF-T piezoelectric composite film exhibited good piezoelectric effect, and thus was applied to several devices. In the seismocardiographic application, the small vibrations generated by the movements of the heart’s aortic valve, mitral valve, and myocardium were converted by the piezoelectric composite film into electrical energy to produce physiological signals. Figure 7a shows the structural design of the seismocardiographic sensor; the black area in the middle is the piezoelectric film and the blue area is the platinum-plated conductive layer. The outermost layer used 3M medical tape as an insulating layer that tightly fit onto the body. Figure 7b shows the three different positions of the film for the seismocardiographic measurements. The red frame indicates the upper part of the pectoralis major muscle near the shoulders, the green frame covers the middle position near the inner side of the pectoralis major muscle, and the blue frame is positioned at the lower edge of the pectoralis major. The seismocardiographs measured at the three different locations are shown in Figure 7c,d for comparison; the upper (red) seismocardiograph was located relatively far from the heart, and consequently, the signal had the most noise and no obvious characteristic peaks; the lower (blue) signal was relatively stable, but there were no obvious atrial valve opening (AO) and mitral valve opening (MO) characteristic peaks; the middle (green) seismocardiographic signal was the most stable and had four distinct characteristic peaks, namely mitral valve opening and closing (MC, MO) and aortic valve opening and closing (AO, AC). According to these results, the CNT–COOH:SMAEF40-M/PVDF-T piezoelectric composite film can be applied to the seismocardiographic sensor. The CNT–COOH:SMAEF40-M/PVDF-T piezoelectric composite film was also used in this study to fabricate a pressure sensor, as shown in Figure 7e. The counterforce from a punch using the boxing glove was harnessed to generate a voltage, and the force of the punch could be calculated through the magnitude of the voltage. This application of the film in a pressure sensor generated considerable sensitivity and output response; furthermore, by using PET film as packaging, the mechanical strength of the film was improved. After continuous punches, the film was not damaged and still delivered good responses. Figure 7f shows the voltage generated by the boxing glove continuously beating against the wall, revealing that the maximum voltage can be as high as 4.8 V. Finally, the piezoelectric composite film was used to prepare energy harvesting elements, as shown in Figure 7g. The CNT–COOH:SMAEF40-M/PVDF piezoelectric composite film was used as a coating on shoes to prepare piezoelectric footwear to collect the voltage generated during walking, which can be stored in an energy storage device. This device can be applied to hiking shoes, snow boots, etc. to provide Global Positioning System (GPS) positioning or send distress signals and to other equipment to provide emergency power. Figure 7h shows the voltages generated by the piezoelectric shoes worn by an adult weighing 65 kg, where the maximum voltage generated was 4.8 V. (Video S1 demonstrates the actual operation of the pressure sensing element). In summary, the CNT–COOH:SMAEF40-M/PVDF-T piezoelectric composite film was successfully applied in a seismocardiographic sensor, a pressure sensor, and an energy harvesting device, demonstrating the considerable commercial potential of the film.

## 4. Conclusions

This study successfully used carbon nanotubes of different dimensions to enhance the crystallinity and β-phase content of PVDF-TrFE for improing the piezoelectric effect of the composite film. The experimental results revealed that modified carbon nanotubes greatly improved the piezoelectric effect of the film. As carbon materials are prone to agglomeration, polymeric dispersants were added to increase the dispersibility of the carbon materials in PVDF-TrFE; moreover, van der Waals forces were exploited to inhibit the stacking and agglomeration of carbon nanomaterials, thereby improving the dispersibility and stability of the carbon materials in organic solvents. The developed composite films also successfully achieved a high voltage of 2.7 V under an applied pressure of 400 N, which was finally applied to seismocardiographic (SCG) sensors, pressure sensors, and energy harvesting elements. In SCG physiological signal sensing, there were obvious characteristic peaks in the seismogram obtained by measuring physiological signals such as the contraction of heart valves and myocardium. In the case of the pressure sensor, the voltage generated by the counterforce from a punch with the boxing glove can be used to calculate the force of the punch, where the maximum voltage reached 4.8 V. Finally, the energy harvesting element can be used in hiking shoes to store the voltage generated during walking for emergency use, where the maximum voltage was 4.8 V. The results indicate that as a result of the van der Waals forces, the β-phase content of the CNT–COOH:SMAEF40-M/PVDF-T piezoelectric composite film increased from 73.6% to 86.4%, which in turn increased the piezoelectric coefficient from 19.8 ± 1.0 to 26.4 ± 1.3 pC/N. This composite film, with its good piezoelectric coefficient, obtained satisfactory performance in three applications, which is ample evidence of the great commercial potential of the CNT–COOH:SMAEF40-M/PVDF-T piezoelectric composite film.

## Figures and Tables

**Table 1:** Measurement results of the crystallinity, β-phase percentage, and piezoelectric coefficient of PVDF-TrEE after different annealing and polarization treatments.

Sample Name	PVDF-TrFE	PVDF-TrFE 135 °C	PVDF-TrFE 145 °C						PVDF-TrFE 155 °C
Applied voltage (V)	0	0	0	1000	2000	3000	4000	5000	0
β/β_0_	—	91.1%	131.7%	—	—	—	—	—	23.9%
Crystallinity	56.8%	70.4%	71.9%	—	—	—	—	—	61.8%
F(β)	62.1%	66.8%	69.5%	70.4%	70.8%	71.3%	73.6%	73.6%	55.6%
d33 (pC/N)	1.8 ± 0.6	—	2.3 ± 0.8	—	—	—	19.8 ± 1.0	—	—

**Table 2:** Measurement results of the crystallinity, β-phase percentage, and piezoelectric coefficient of nanocarbon/PVDF-T.

Sample	Dispersant	Content (wt%)	Crystallinity (%)	*F(*β*)* (%)	d_33_ (pC/N)
OCB/PVDF-TrFE	—	0.1	78.9	74.4	—
	—	0.5	80.6	79.6	—
	—	1.0	83.1	80.9	23.2 ± 1.4
	—	3.0	82.4	78.3	—
	—	5.0	81.2	74.3	—
CNT–COOH/PVDF-TrFE	—	0.1	82.1	75.2	—
	—	0.5	82.9	77.9	—
	—	1.0	83.4	82.8	24.3 ± 1.3
	SMA 1000	1.0	83.5	84.2	—
	SMA 2000	1.0	83.6	85.1	—
	SMA EF40	1.0	83.8	86.4	26.4 ± 1.3
	SMA EF80	1.0	83.4	84.5	—
	—	3.0	81.8	76.6	—
	—	5.0	79.7	74.7	—
GO/PVDF-TrFE	—	0.1	73.0	74.8	—
	—	0.5	75.2	75.7	—
	—	1.0	75.4	76.5	—
	—	3.0	76.6	77.3	20.6 ± 1.3
	—	5.0	75.5	76.8	—

**Table 3:** Continuous output voltage results of PVDF-TrFE, CNT–COOH/PVDF-T 1 wt%, and CNT–COOH:SMA EF40-M/PVDF-T 1:1.

Sample Name	200 N		300 N		400 N	
	Max Voltage (V)	Avg. Voltage (V)	Max Voltage (V)	Avg. Voltage (V)	Max Voltage (V)	Avg. Voltage (V)
PVDF-TrFE	0.96	0.71	1.32	1.04	1.66	1.31
CNT–COOH/PVDF-T 1 wt%	1.46	1.22	1.98	1.61	2.50	2.16
CNT–COOH:SMA EF40-M/PVDF-T 1:1	1.57	1.34	2.06	1.75	2.72	2.36

## Supplementary Materials

The following are available online at https://www.mdpi.com/2073-4360/12/12/2999/s1, Figure S1: (a) FTIR curves of PVDF-TrEE polarization at different voltages, (b) hysteresis curve of PVDF-TrEE after annealing treatment at 145 °C, Figure S2: FTIR curves of carbon nanomaterials before and after oxidation: (a) CB, (b) CNT, and (c) GO, Figure S3: DSC (a) cooling and (b) heating curves of PVDF-TrEE and the different carbon nanomaterial/PVDF-T, Figure S4: Schematic diagram illustrating the chemical reaction of the polymeric dispersant, Figure S5: FT-IR spectra of the (a) SMA EF40 and (b) SMA EF40-M polymeric dispersants, Figure S6: The water contact angle of PVDF-TrEE, CNT–COOH/PVDF-T 1 wt% and CNT–COOH:SMA EF40-M/PVDF-T 1:1, Figure S7: The Raman spectroscopy of PVDF-TrEE, CNT–COOH/PVDF-T 1 wt% and CNT–COOH:SMA EF40-M/PVDF-T 1:1.
