# Supplementary material for: Enhanced Piezoelectric Properties of Poly(Vinylidenefluoride-Co-Trifluoroethylene)/Carbon-Based Nanomaterial Composite Films for Pressure Sensing Applications
Source: Polymers (Basel). 2020 Dec 16;12(12):2999. doi: 10.3390/polym12122999 (PMC7765614; doi:10.3390/polym12122999)
Supplement: Supplementary file 1 [file polymers-12-02999-s001.zip › polymers-1039732-support -final.docx]

Supporting Information

Enhanced Piezoelectric Properties of Poly(Vinylidenefluoride-Co-Trifluoroethylene)/Carbon-Based Nanomaterial Composite Films for Pressure Sensing Applications

Jia-Wun Li ^1,†^, Chen-Yang Huang ^1,†^, Kuan-Yu Chen ^1,†^, Jian-Xun Chen ^1^, Xiao-Yong Hsu ^1^,

Yan-Feng Chen ^1^, Chung-Feng Jeffrey Kuo ^1^, Chih-Chia Cheng ^2^, Maw-Cherng Suen ^3^

and Chih-Wei Chiu ^1,^*

^1^ Department of Materials Science and Engineering, National Taiwan University of Science and Technology, Taipei 10607, Taiwan; [a12352335@yahoo.com.tw](mailto:a12352335@yahoo.com.tw) (J.-W.L.); [D10504015@mail.ntust.edu.tw](mailto:D10504015@mail.ntust.edu.tw) (C.-Y.H.); [M10704205@mail.ntust.edu.tw](mailto:M10704205@mail.ntust.edu.tw) (K.-Y.C.); [ch60210@gmail.com](mailto:ch60210@gmail.com) (J.-X.H.); [xyhsu.0879@ttri.org.tw](mailto:xyhsu.0879@ttri.org.tw) (X.-Y.H.); [kk0960216886@gmail.com](mailto:kk0960216886@gmail.com) (Y.-F.C.); [jeffreykuo@mail.ntust.edu.tw](mailto:jeffreykuo@mail.ntust.edu.tw) (C.-F.J.K.)

^2^ Graduate Institute of Applied Science and Technology, National Taiwan University of Science and Technology, Taipei 10607, Taiwan; [cccheng@mail.ntust.edu.tw](mailto:cccheng@mail.ntust.edu.tw)

^3^ Department of Fashion Business Administration, Lee-Ming Institute of Technology,
New Taipei City 24305, Taiwan; [sunmc0414@gmail.com](mailto:sunmc0414@gmail.com)

***** Correspondence: cwchiu@mail.ntust.edu.tw; Tel.: +886-2-2737-6521; Fax: +886-2-2737-6544

† These authors contributed equally to this work.

Received: 30 November 2020; Accepted: 14 December 2020; Published: date


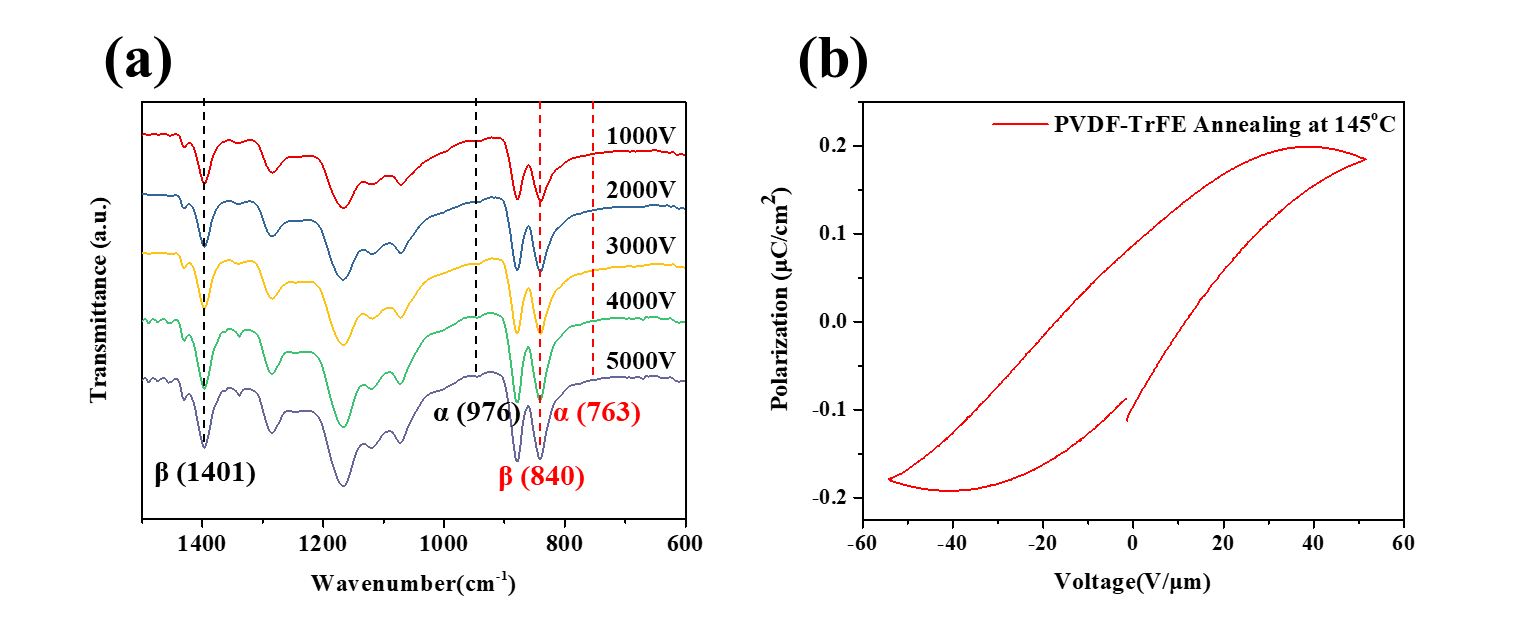


**Figure S1.** (**a**) FTIR curves of PVDF-TrEE polarization at different voltages, (**b**) hysteresis curve of PVDF-TrEE after annealing treatment at 145 °C.


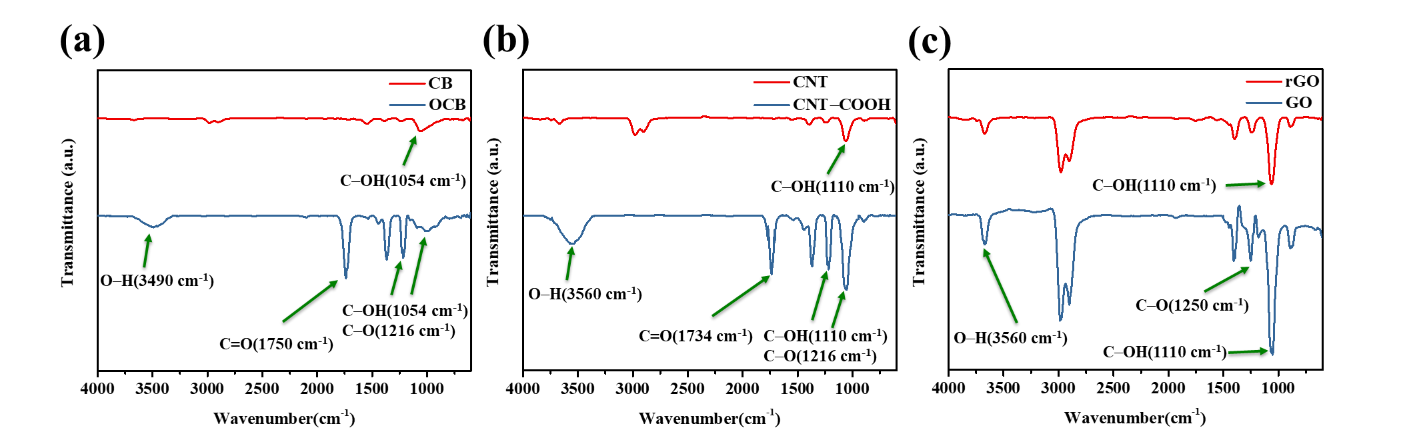


**Figure S2.** FTIR curves of carbon nanomaterials before and after oxidation: (**a**) CB, (**b**) CNT, and (**c**) GO.


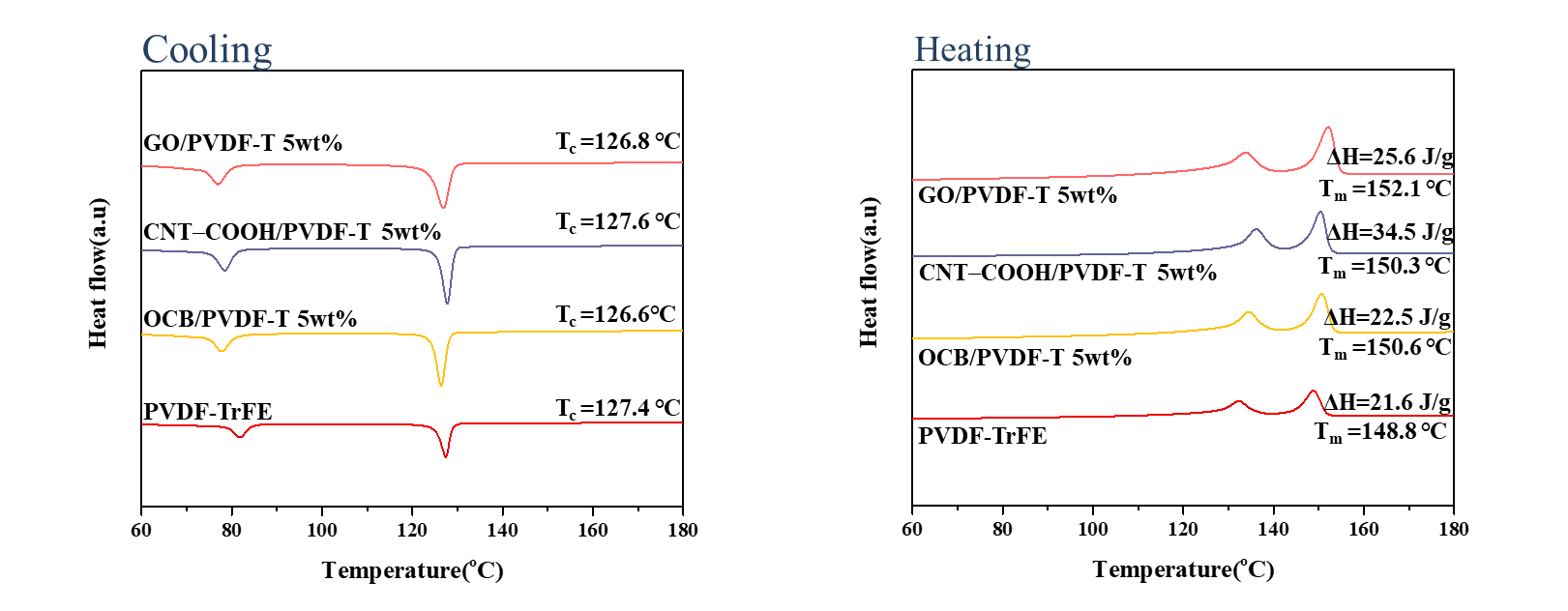


**Figure S3.** DSC (**a**) cooling and (**b**) heating curves of PVDF-TrEE and the different carbon nanomaterial/PVDF-T.


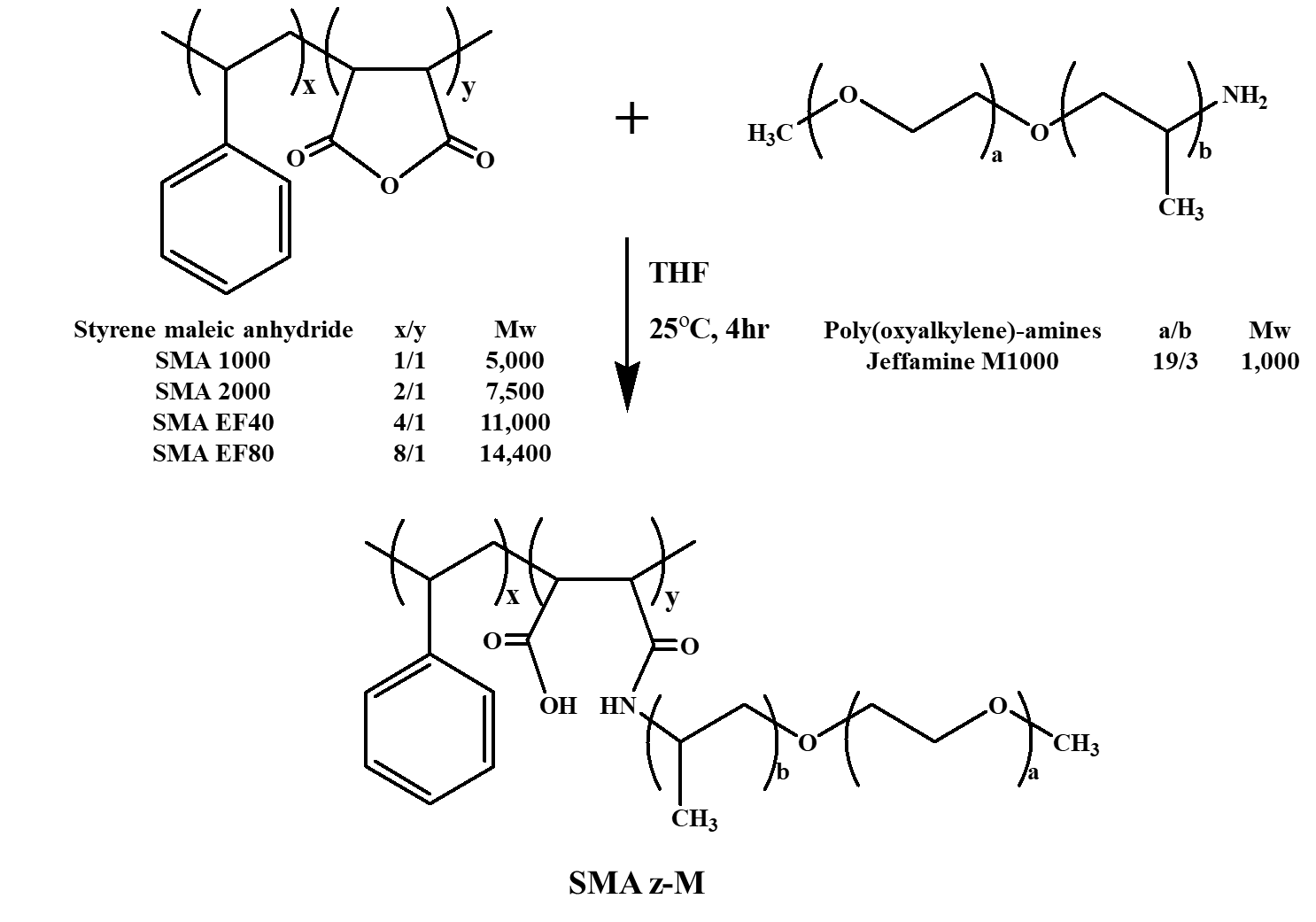


**Figure S4.** Schematic diagram illustrating the chemical reaction of the polymeric dispersant.


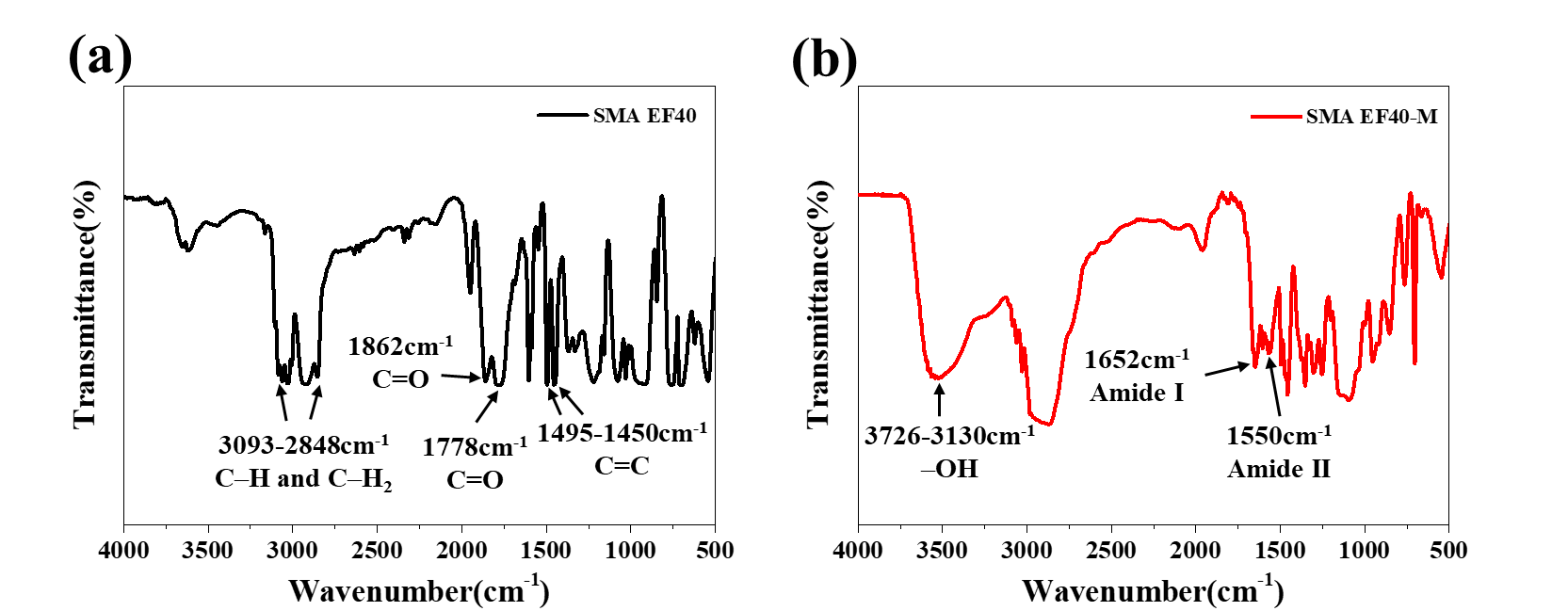


**Figure S5.** FTIR spectra of the (**a**) SMA EF40 and (**b**) SMA EF40-M polymeric dispersants.


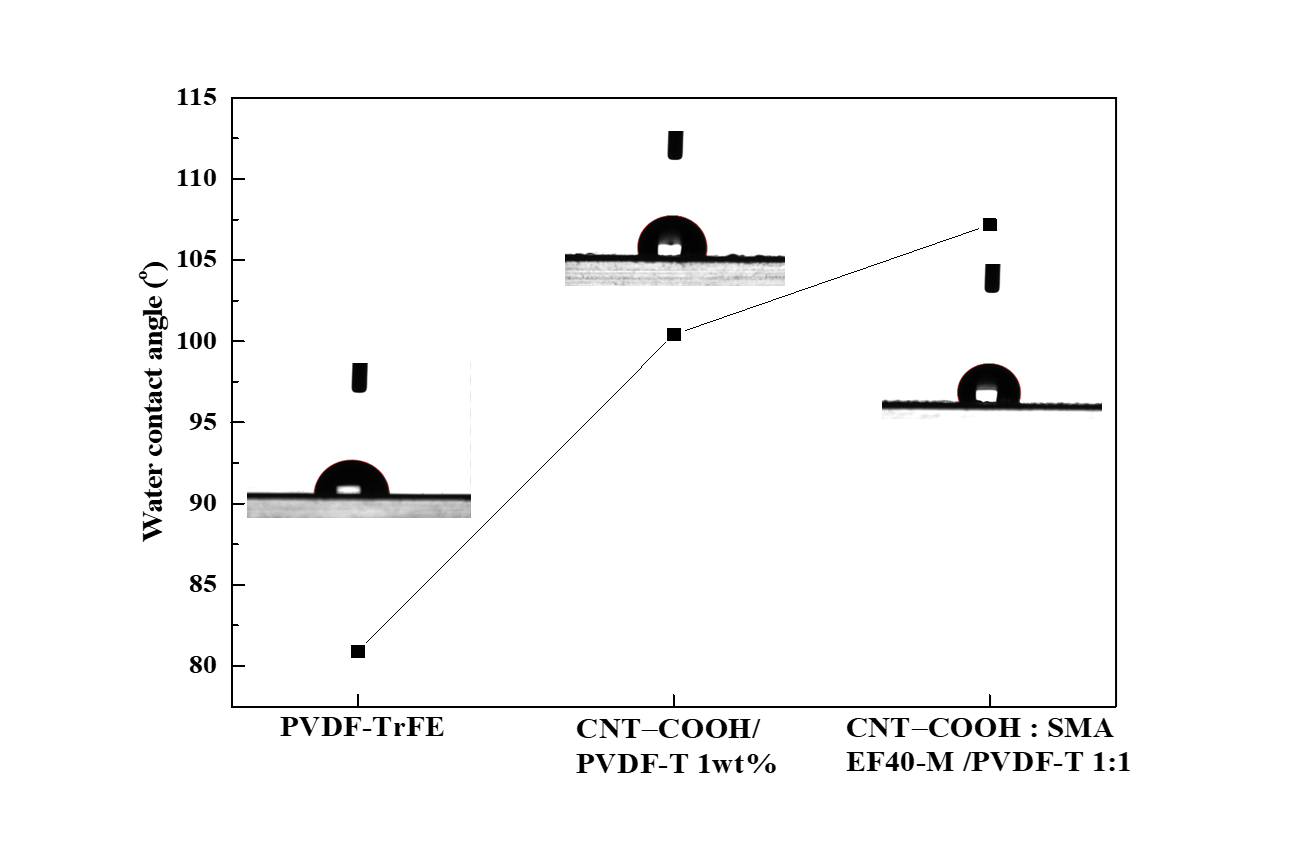


**Figure S6.** The water contact angle of PVDF-TrEE, CNT-COOH/PVDF-T 1 wt% and CNT–COOH: SMA EF40-M/PVDF-T 1:1.


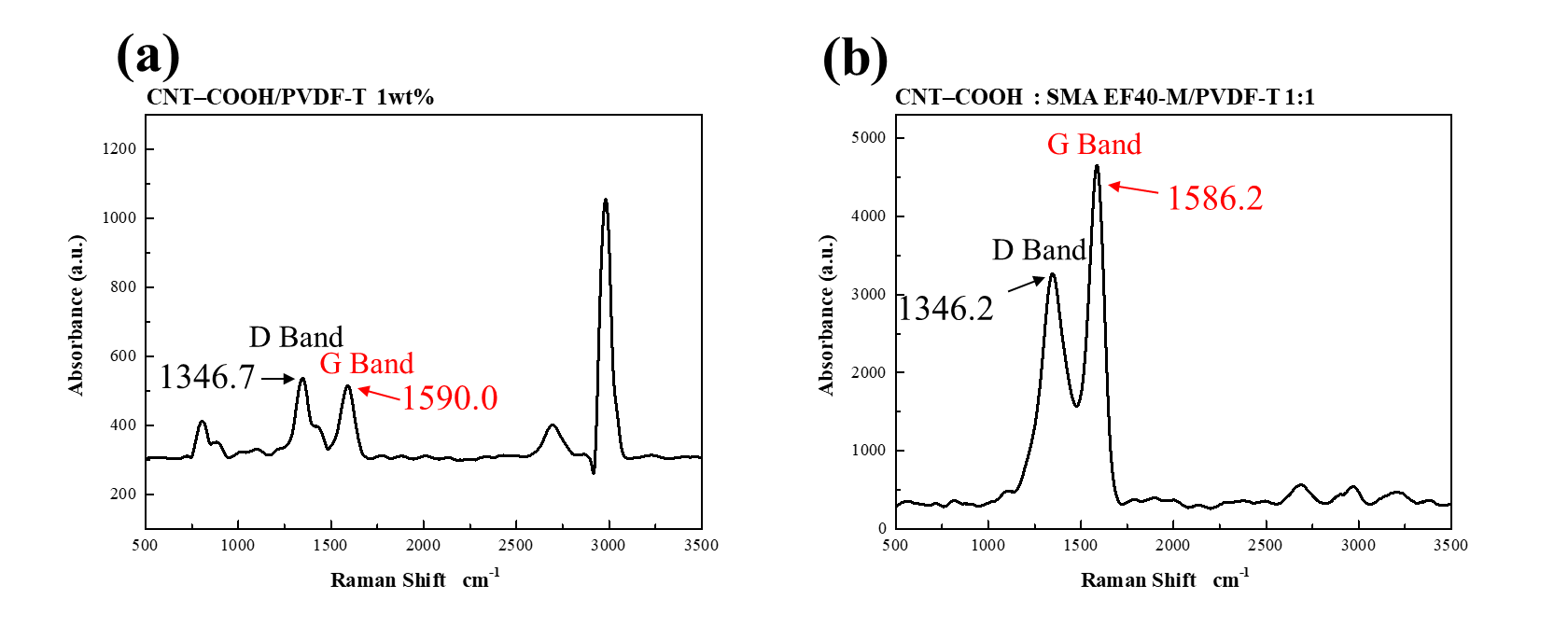


**Figure S7.** The Raman spectroscopy of PVDF-TrEE, CNT–COOH/PVDF-T 1 wt% and CNT–COOH: SMA EF40-M/PVDF-T 1:1.
